# The Anti-Inflammatory Properties of Mesenchymal Stem Cells in Epilepsy: Possible Treatments and Future Perspectives

**DOI:** 10.3390/ijms21249683

**Published:** 2020-12-18

**Authors:** Valentina Salari, Francesca Mengoni, Federico Del Gallo, Giuseppe Bertini, Paolo Francesco Fabene

**Affiliations:** Department of Neurosciences, Biomedicine and Movement Sciences, School of Medicine, University of Verona, 37134 Verona, Italy; valentina.salari@univr.it (V.S.); francesca.mengoni@univr.it (F.M.); federico.delgallo@univr.it (F.D.G.); giuseppe.bertini@univr.it (G.B.)

**Keywords:** neurological disorders, epileptogenesis, inflammation, epileptic seizure, cell therapy

## Abstract

Mesenchymal stem cells (MSCs) are multipotent adult cells with self-renewing capacities. MSCs display specific properties, such as the ability to repair damaged tissues, resulting in optimal candidates for cell therapy against degenerative diseases. In addition to the reparative functions of MSCs, growing evidence shows that these cells have potent immunomodulatory and anti-inflammatory properties. Therefore, MSCs are potential tools for treating inflammation-related neurological diseases, including epilepsy. In this regard, over the last decades, epilepsy has no longer been considered a purely neuronal pathology, since inflammatory events underlying the genesis of epilepsy have been demonstrated. This review assessed current knowledge on the use of MSCs in the treatment of epilepsy. Mostly, attention will be focused on the anti-inflammatory and immunological skills of MSCs. Understanding the mechanisms by which MSCs might modulate the severity of the disease will contribute to the development of new potential alternatives for both prophylaxis and treatment against epilepsy.

## 1. Mesenchymal Stem Cells

Mesenchymal stem cells (MSCs) are multipotent adult stem cells with self-renewal abilities, which can differentiate into different lineages of mesenchymal tissue, including chondrocytes, osteoblasts, and adipocytes [1,2]. Studies suggest that under certain conditions, both experimental and physiological, MSCs may also be able to differentiate into further cell types, including neurons, cardiomyocytes, and endothelial cells [3,4,5]. MSCs can be found in almost all tissues, but the most well-known sources of MSCs are the bone marrow and the vascular fraction of adipose stroma; in recent decades, the placenta and the umbilical cord have also been identified as excellent sources of MSCs [6]. Whatever the source, MSC collection does not raise ethical concerns and cultures can be reliably obtained and maintained [7]. MSCs are characterized by a fibroblast-like morphology and are easily distinguished from hematopoietic stem cells thanks to their strong adherence to standard culture dishes and their marker expression pattern. Specifically, they are generally positive for CD90, CD73, and CD105 and negative for the hematopoietic markers CD45, CD34, CD79a or CD19, CD14 or CD11b, and human leukocyte antigen DR [8].

Although the in-vitro phenotype of MSCs is well-known, understanding their physiology in vivo has been made difficult by the lack of specific markers. Recent advances in murine research, however, may provide new tools for evaluating the role of MSCs in physiological conditions. Specifically, recent studies have demonstrated that MSCs provide a perivascular niche for hematopoietic stem cells and regulate their trafficking, along with those of immune cells. It has also been suggested that the MSC function depends on the tissue context and molecular signals, which could induce MSCs to release cytokines and chemokines, with either pro-inflammatory or anti-inflammatory effects [1,9,10].

## 2. MSCs and Mechanisms of Action

MSCs have been regarded as promising agents for regenerative medicine, due to their self-renewal and multilineage differentiation capacity [11,12]. In recent years, in-vitro studies have shown that, besides their ability to differentiate into distinct cell types, MSCs may also exert therapeutic effects by cell “enhancement”, through the release of trophic and anti-inflammatory factors, which may re-establish the physiological environment. Indeed, MSCs also have a modulatory role in the inflammatory and immune response. Based on these features, MSCs have been employed as therapeutic tools in a variety of clinical conditions other than regenerative medicine, including autoimmune and inflammatory disorders [13,14,15,16,17].

**Figure 1:**
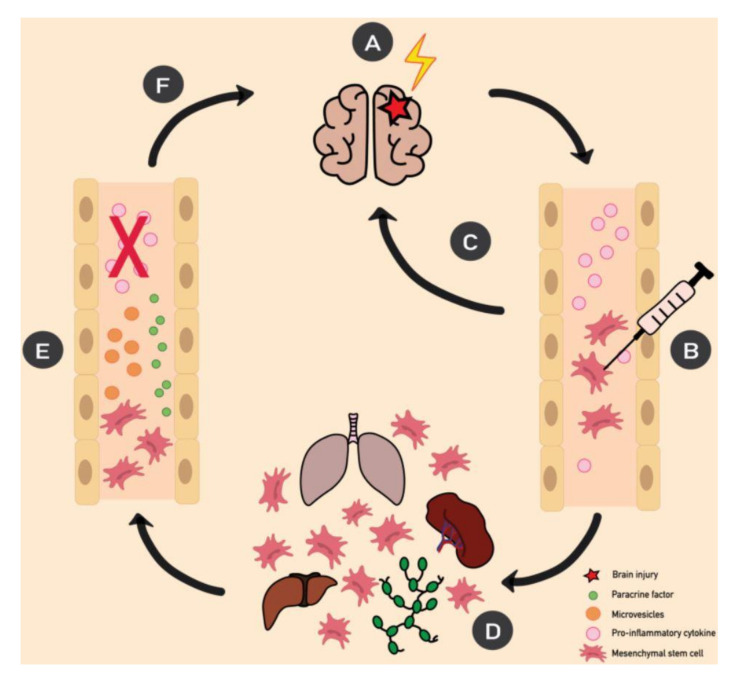
Schematic of the putative mechanisms of action of mesenchymal stem cell (MSC) therapy in neurological diseases. (**A**) Neurological challenges, such as infections, head trauma, and autoimmune diseases, may lead to brain injury, characterized by a release of proinflammatory cytokine. However, even peripheral organ damage may upregulate the levels of proinflammatory cytokines in circulating blood, finally reaching the brain throughout systemic circulation. Once circulating cytokines interact with a neuro-vascular unit, the neuronal excitability threshold is lower and electrical paroxysmal events may happen, leading to a possible alteration of the local neuronal circuitry. (**B**) When MSCs are administered peripherally, (**C**) a smaller part is directly mobilized to the site of injury, while (**D**) most cells get trapped in organs such as the liver, lungs, lymph nodes, and spleen. (**E**) Here, MSCs are activated by inflammatory mediators and release paracrine factors and microvesicles in the bloodstream. (**F**) These molecules reach the site of injury, and, together with MSCs previously recruited, modulate the progression of inflammation and facilitate tissue repair.

A large body of work shows that, following cell or tissue injury, MSCs can activate by inflammatory cytokines, move to the damage site, and control the tissue-regeneration process by releasing an array of factors that may facilitate the differentiation and proliferation of progenitor cells while inhibiting inflammatory responses [18]. The secreted factors include cytokines, such as interleukin (IL)-10 and growth factors (GF), including stromal cell-derived factor-1 (SDF-1), epidermal GF (EGF), keratinocyte GF (KGF)-1, fibroblast GF (FGF), vascular endothelial GF (VEGF), plated-derived GF (PDGF), hepatocyte GF (HGF), transforming growth factor (TGF)-β, and insulin GF (IGF)-1 [19]. The exact mechanisms by which MSCs are recruited and activated at the damage site are still unclear (Figure 1) [20,21].

We do know that most peripherally-administered MSCs reach, and are potentially trapped in, organs such as the spleen, lungs, liver, and lymph nodes, making the rates of MSC engraftment quite poor [22,23]. Recent evidence has shown the effectiveness of secretome- or MSC-conditioned medium (CM) in preclinical studies in view of its therapeutic potential in pathologies such as gastric mucosal injury, colitis, and cardiovascular diseases [24,25,26] Moreover, it has been demonstrated that MSCs are also capable of releasing exosomes and microvesicles, which could have biological properties similar to those possessed by whole MSCs. Therefore, MSC-derived extracellular vesicles could be used as an alternative MSC-based therapy with a superior safety profile [27]. Taken together, the evidence points to a hypothetical paracrine mechanism for anti-inflammatory and immunomodulatory MSCs’ properties [28,29].

## 3. MSC and Mechanisms of Action in Inflammation

Recently, significant advances have been made in understanding the mechanisms underlying the immunomodulatory and anti-inflammatory properties of MSCs. In-vitro studies show that MSCs may respond in opposite directions, depending on the intensity of environmental signals. Namely, they appear to promote inflammation when the immune system is underactivated and suppress it when the immune system is overactivated [30,31]. At the early stages of inflammation, MSCs sense pro-inflammatory signals through receptors for IL-1β, interferon (IFN)-γ, Toll-like receptors, and tumor necrosis factor (TNF)-α, and enhance inflammation. They promote T-cell activation by secreting chemokines, including the C-X-C motif ligand (CXCL)9, macrophage inflammatory protein-1, CCL5, and CXCL10, and recruiting more lymphocytes. At this stage, the low levels of inflammatory signals, such as TNF-α and IFN-γ, are sufficient to upregulate their chemokine secretion, but not to induce a significant expression of immunomodulatory mediators, such as inducible nitric oxide synthase (iNOS) in mice or indoleamine 2,3-dioxygenase (IDO) in humans [32].

At later stages, higher levels of pro-inflammatory factors, such as IL-1β, IFN-γ, and TNF-α, stimulate MSCs to reduce inflammation and avoid autoimmune reactions by releasing, for example, TGF-β, IL-10, IDO, or iNOS, resulting in an inhibition of the migration, maturation, and antigen presentation of dendritic cells (DC), and of T-cell function and proliferation [33,34], along with the proliferation of regulatory T cells [30,35]. Therefore, the IDO or iNOS level has been proposed as the switcher between the pro- and anti-inflammatory effects of MSCs [36].

Moreover, MSCs have been shown to provide in-vitro immunosuppressive activities on activated B cells. Specifically, MSCs decrease B-cell proliferation by arresting their cell cycle and affect B-cell differentiation, as immunoglobulin (Ig) M, IgG, and IgA production are diminished [37]. A wealth of data suggests the role of MSCs in the induction of regulatory B cells [38]. More recent evidence shows that human MSC-treated regulatory CD23+ CD43+ B cells erase intestinal inflammation [39] and human umbilical cord-derived MSCs defend against colitis via CD5+ regulatory B cells [40]. Therefore, MSCs may suppress inflammation processes in different ways, through the upregulation of anti-inflammatory factors or the downregulation of proinflammatory factors. In addition, MSCs may also suppress immune reactions via direct cell contact.

## 4. Epilepsy

Epilepsy is a chronic neurological condition affecting over 50 million people worldwide, with about 5 million people diagnosed with epilepsy every year [41,42]. Epilepsy is defined as a brain pathology characterized by recurrent, unprovoked epileptic seizures and is related to a variety of neurological diseases and other medical conditions [43]. It is estimated that around 60% of patients diagnosed with epilepsy have focal epilepsy, and among these, temporal lobe epilepsy (TLE) is the main cause of refractory epilepsy [44].

Although both environmental and genetic factors contribute to individual predisposition to epilepsy, in most cases, its physiopathology is still unknown [45]. Nowadays, the first-line therapeutic approach is pharmacological, based on anti-seizure medications that act by reducing the neuronal excitability, either through an inhibition of sodium ion channels or by enhancing the activity of γ-aminobutyric acid (GABA) receptors. However, almost 30% of patients are refractory to these therapies. Additionally, anti-seizure medications may have side effects in several patients, and provide a purely symptomatic treatment, without acting on epileptogenesis mechanisms [46,47]. For these reasons, it is extremely important to develop alternative therapeutic approaches.

Recently, new experimental treatment approaches have been explored, including microRNA silencing [48], gene therapy, and neural cell transplantation. In particular, the goal of the latter two approaches is to recreate damaged circuits, enhance the inhibitory response through the replacement of GABAergic neurons, and increase anti-inflammatory mediators and trophic factors respectively produced by microglia and astrocytes [49]. However, these approaches have limitations, including a delay in gene expression after transfection or the need to perform transplantation at multiple sites, given the multiple brain areas where an epileptic status can occur. An alternative method would be to use pluripotent stem cells; however, these are not yet ready to be handled in clinical studies, given the high probability of developing a teratoma and triggering immunological complications [50].

MSC transplantation, on the other hand, appears to be a promising treatment for some neurological disorders, including epilepsy [51]. Indeed, some of the distinctive features of MSCs make them excellent candidates for this purpose. These properties include a high homing potential, a limited probability of developing teratomas, renewal and regeneration expertise and, most importantly, anti-inflammatory properties [52]. In particular, the immunomodulatory and anti-inflammatory properties of MSCs represent an opportunity for future research on the treatment of epilepsy. In the last few years, evidence from both preclinical and clinical studies has suggested that inflammation might be both a consequence and a cause of epilepsy [53].

## 5. MSCs and Epilepsy

Due to their intrinsic properties, MSCs have been proposed as a safe and promising asset for the treatment of epilepsy in the pre-clinical setting [54]. In most cases, refractory temporal lobe epilepsy is associated with hippocampal sclerosis, aberrant mossy fiber sprouting, and the loss of neurons. Therefore, MSCs were initially tested to treat epilepsy by repairing and/or regenerating damaged tissues. In particular, several preclinical studies were carried out to test the ability of MSCs to reduce epileptogenesis, which is the process by which a brain network is functionally altered by an increased seizure susceptibility, thus enhancing the probability to generate spontaneous recurrent seizures [55]. In these studies, MSCs were injected in several animal models of epilepsy, either intravenously or directly in the hippocampus. While the results were sometimes comparable (see below), some studies suggested that MSCs have the best therapeutic effect when they are directly injected in the central nervous system [56].

An intravenous infusion of MSCs in a rat lithium-pilocarpine injection model of temporal lobe epilepsy has been shown to reduce epileptogenesis and preserve cognitive function by inhibiting neuronal cell death and decreasing aberrant mossy fibers of the hippocampus [54]. Further studies have shown that the intravenous administration of bone marrow stromal cells reduced the number of seizures and preserved neurons from death and neurophagia in a rat pilocarpine or lithium-pilocarpine model of TLE [57,58,59]. Using the same animal model, MSCs injected in the right hippocampus reduced the amplitude and frequency of electroencephalographic spike-waves and improved the dysregulated adenosine receptor expression that accompanied experimentally-induced epilepsy in rats [60]. Similarly, intra-hippocampal injections of human umbilical mesenchymal stem cells (HUMSCs) attenuated the incidence and duration of spontaneous recurrent seizures in a rat pilocarpine TLE model. The mechanisms underlying these effects include a reduction of mossy fiber sprouting, a decrease of neuron and interneuron loss, and the suppression of *status epilepticus* (SE)-induced brain inflammation [61].

Another study revealed that the intranasal administration of extracellular MSC-derived vesicles reduced SE-induced neuroinflammation, cognitive dysfunction, and aberrant neurogenesis in a mouse pilocarpine model of epilepsy [62]. In-vitro studies have shown that the ability of MSCs to reduce epileptogenesis is partially due to their capacity to protect neurons against glutamate excitotoxicity. It has been suggested that this protection is associated with a reduced expression of N-methyl-D-aspartate receptor subunits and glutamate-induced Ca^2+^ responses [63].

Additionally, MSC culture medium decreases levels of the GluR1 subunit of the AMPA receptor on the neural cell surface, thus reducing neuronal glutamate signaling [64].

Genetically engineered bone marrow MSCs have also been proposed: The silencing of Hes1, which is a gene involved in the neural differentiation of bone marrow stem cells, has been proven to promote the differentiation of these cells into GABAergic neuron-like cells, improving the functional outcome in a rat model of epilepsy [65].

The silencing of Hes1 in bone marrow stem cells has been shown to promote their differentiation into GABAergic neuron-like cells [65]. The use of genetically engineered bone marrow MSCs in a lithium-pilocarpine rat model of epilepsy resulted in a functional improvement. Benefits were proportional to the degree of cell differentiation [65].

Currently, only a single clinical study regarding the use of MSCs in epilepsy treatment has been published. This study, not registered on NIH ClinicalTrials.gov, demonstrated that therapy with autologous MSC decreases the number of seizures in electroencephalography evaluation and leads to cognitive improvements in children with drug-resistant epilepsy. Moreover, the results do not show early or late side effects associated with the transplantation procedure or the therapy itself [66].

## 6. Role of Inflammation in the Pathogenesis of Epilepsy

**Figure 2:**
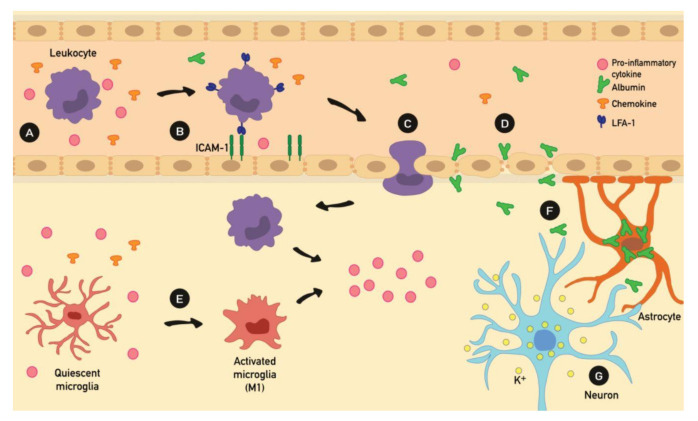
Cascade of events during inflammation-mediated seizures. (**A**) An initiating inflammatory challenge causes an increase of proinflammatory mediators such as cytokines and chemokines, (**B**) which in turn leads to an upregulation of leukocyte ligands (e.g., LFA-1) and endothelial counterligands (e.g., ICAM-1), eventually resulting in leukocyte recruitment. (**C**) Upregulation of adhesion molecules reflected in leukocyte migration into the brain parenchyma and (**D**) a morphological change in the endothelial cells’ shape, finally resulting in an alteration of tight junctions; these processes will lead to blood–brain barrier (BBB) leakage with the extravasation of serum components (e.g., albumin). (**E**) Microglial cells are consequently activated by proinflammatory cytokines. (**F**) Moreover, the albumin extravasation induces a decrease of inward-rectifying K^+^ channels in astrocytes, leading to an increase of extracellular potassium in brain parenchyma. (**G**) Therefore, the higher extracellular K^+^ level facilitates neuronal hyperexcitability and induces seizure activity.

Historically, epilepsy was considered a “neuron-centered” disease. Recent studies have revealed that the neuronal activity itself depends on the close interactions between neurons, glial cells, the blood–brain barrier (BBB) permeability, the vascular endothelium, immune-related blood cells, and a “humoral network” of diffusible factors that includes various cytokines and chemokines. From this perspective, the neurovascular unit is regarded as an extremely important complex for understanding the pathogenesis of epilepsy [67], especially with regards to the recently emerged role of inflammation in epileptogenesis. A growing body of literature suggests that inflammatory mechanisms play an important role in the development and progression of seizure activity. Conversely, seizures can induce inflammation of the brain and their recurrence may lead to chronic inflammation (Figure 2) [68].

Either prolonged or focal febrile seizures in childhood have been associated with an increased risk of developing an intractable form of temporal lobe epilepsy [69]. Another clinical study showed that febrile seizures raise the levels of pro-inflammatory cytokines IL-6, TNF-α, and IL-1β in cerebrospinal fluid [70]. Furthermore, human brain tissue obtained during the surgical resection of the seizure focus in patients with refractory focal epilepsy expressed significantly more biomarkers of inflammation than samples from non-epileptic patients [71].

In-vitro and in-vivo research models allow in-depth investigations of the inflammatory processes that can trigger (or be triggered by) seizure activity. Increasing evidence supports the existence of a complex interplay between systemic inflammatory processes and brain homeostasis. For example, autoimmune diseases or common infections can predispose to spontaneous recurrent seizures [72]. Interestingly, when the lipopolysaccharide, which is a component of Gram-negative bacteria, was administered chronically in rats, an increased permeability of the BBB was observed as a consequence of the systemic inflammation [73]. Therefore, the interplay between the peripheral immune response and the central nervous system may play a role in the development of seizures and ultimately, epilepsy.

Nevertheless, an increasing number of clinical and preclinical studies have revealed that inflammation may play a role in seizure susceptibility, even without immune-mediated pathologies or infections. An increase in TNF, IL-6, IL-1β, and the IL-1 receptor antagonist has been found in blood serum and cerebrospinal fluid in patients with chronic epilepsy after seizures [74]. Similarly, VEGF, TGF-β1, IL-6, IL-1β, and TNF-α upregulated their mRNA expression in the hippocampus after seizures [75].

## 7. BBB Permeability and Inflammation in Epilepsy

Increasing evidence, from both research models and human specimens, suggests that leukocyte–endothelial interaction mechanisms may play an important role in the pathogenesis of epilepsy. It was shown that leukocyte trafficking mechanisms promote BBB leakage, which led to seizure activity [76]. Moreover, patients with different types of epilepsy exhibited higher numbers of leukocytes in brain parenchyma compared to control patients [77]. Along with leukocyte migration through BBB, albumin extravasation has been suggested to be involved in epileptogenesis.

Indeed, extravasated albumin was found to induce epileptogenesis binding TGF receptor 2 in astrocytes. This results in astrocytic transformation and dysfunction, characterized by a downregulation of inward-rectifying K^+^ channels, interfering with the physiological buffering of extracellular K^+^ ions by astrocyte. Therefore, the increased availability of K^+^ ions in the extracellular compartment facilitates neuronal hyperexcitability, and eventually induces epileptiform activity [78,79].

## 8. MSCs and Inflammation in Epilepsy

**Figure 3:**
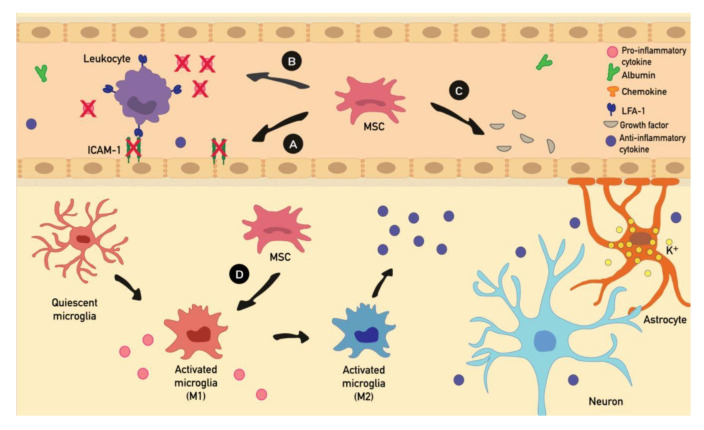
The putative effects of MSC therapy on the inflammatory cascade of events during seizures. (**A**) MSCs downregulate adhesion molecule (e.g., ICAM-1) expression. (**B**) MSCs inhibit leukocytes by blocking their pro-inflammatory factor expression, resulting in decreasing levels of proinflammatory cytokine and leukocyte cell recruitment at the site of damage. (**C**) MSCs release various factors (e.g., KGF, EGF, VEGF and PDGF), involved in maintaining endothelial integrity and preventing interactions between leukocytes and endothelial cells. (**D**) In addition, MSCs promote the switching of microglia from a pro-inflammatory (type 1) to an anti-inflammatory (type 2) phenotype, thus increasing the level of anti-inflammatory cytokines (IL-4 and IL-10). Anti-inflammatory cytokines reduce inflammation by suppressing microglia and T cell activation, pro-inflammatory cytokine production, and leukocyte recruitment.

Cell therapies with MSCs have been proposed as a possible treatment for inflammatory diseases [16,17]. This has suggested that, in addition to neuroprotection and the reshaping of neuronal circuits, the therapeutic mechanism of MSCs in treating epilepsy might also include immunomodulation and an anti-inflammatory pathway. As mentioned above, seizures can cause brain damage and consequently, a loss of neurons, but not all seizures seem to cause structural lesions detectable by magnetic resonance imaging [80]. On the other hand, epileptic seizures cause an inflammatory response and a consequent immunological reaction [81]. Therefore, MSCs are likely to act via the release of cytoprotective, immunomodulatory, and anti-inflammatory factors that can contribute to the recovery and rebalancing of damaged tissues (Figure 3) [36].

An intravenous administration of bone marrow mononuclear cells (BMMCs), which is a heterogeneous population comprised of both stem cells and progenitor cells with potential therapeutic value, in an animal model of TLE, resulted in a decrease of cell death and inflammatory mediators, without any differentiation of BMMCs into neurons [82]. Since the success of MSC-based therapy does not correlate with the efficiency of cell replacement and engraftment, it is thought that the anti-inflammatory and neuroprotective MSCs’ properties are mainly exercised throughout paracrine mechanisms [83,84]. However, since MSCs were found in the hippocampus after intravenous administration, it is also possible that MSCs migrate directly into the brain to exert their effect [54].

As previously mentioned, evidence from both preclinical animal models and clinical studies suggests that inflammation might be both a consequence and a cause of epilepsy. Moreover, epileptic seizures generate a pro-inflammatory *milieu* by raising the levels of IL-6, TNF-α, and IL-1β in the hippocampus and cerebrospinal fluid [70,85]. This environment attracts MSCs at the sites of injury where they are licensed to exert their immunomodulatory purposes. Therefore, MSCs could respond to excessive proinflammatory signals, such as through their receptors for IL-1β, IFN-γ, and TNF-α. To exercise their anti-inflammatory and immunosuppressive functions, MSCs need to be stimulated by IFN-γ, in combination with one (or more) proinflammatory cytokines (TNF-α, IL-6, IL1-β, or IL-1α). Therefore, MSCs could respond to excessive proinflammatory signals, by releasing a burst of anti-inflammatory cytokines, chemokines, and a high level of immunosuppressive factors [32].

It has been reported that, in animal models of epilepsy, BMMC therapy increased the level of anti-inflammatory cytokines (IL-4 and IL-10) and decreased proinflammatory cytokine levels (IL-6, Il-1β, and TNF-α) in the brain and blood serum [82]. Moreover, bone marrow mononuclear cell transplantation reduced gliosis and neural loss [58].

MSCs may release various factors, such as Angiopoietin 1, VEGF, HGF, KGF, EGF, PDGF, FGF, and TGF-β, which directly influence endothelial cells. These paracrine trophic factors are important in maintaining endothelial integrity and promoting angiogenesis through the regulation of endothelial cell proliferation, reduction of endothelial permeability, and prevention of interactions between leukocytes and endothelial cells [86].

### 8.1. MSCs and Lymphocytes

As previously stated, leukocyte–endothelial interaction has been demonstrated to play a key role in the epileptogenic process. The inhibition of leukocyte–vascular interactions markedly reduced seizures in a mouse model of TLE. Lymphocytes have been demonstrated to be the principal actors of the inflammatory response, even if neutrophil depletion can also be protective [76]. Regarding lymphocytes, the Th1, but not Th2, phenotype appears to be recruited at the site of injury after seizures. This indicates that Th1 immune cells play a key role in the induction of inflammation, thus contributing to the progression of epilepsy [76].

MSCs are known to induce the differentiation and maturation of Th2 cells through IDO expression, resulting in the depletion of tryptophan and the accumulation of its metabolites, which eventually induce Th1 cell apoptosis [87]. Moreover, MSC inhibits Th1 cells directly by blocking their pro-inflammatory factor expression, or indirectly through the suppression of DC and NK cells [88]. This evidence might suggest that one of the mechanisms by which MSCs may suppress inflammation in epilepsy is the decrease of Th1 cell recruitment at the site of damage.

Recent studies have revealed that the blockage of leukocyte–vascular adhesion prevented BBB disruption in animal models of epilepsy [76]. It has been demonstrated that MSCs are able to downregulate intercellular adhesion molecule 1 (ICAM-1) expression both in vivo and in vitro [89]. ICAM-1 is an endothelial cell adhesion molecule directly involved in sustaining leukocyte adhesion and BBB integrity; a downregulation of ICAM-1 activation has been demonstrated to play a protective role in epileptogenesis [76]. Currently, it is still unclear which factors are secreted by MSCs to decrease ICAM-1 expression in endothelial cells.

### 8.2. MSC and Glial Cells

In addition, MSCs promote the switching of macrophages from a pro-inflammatory (type 1) to an anti-inflammatory (type 2) phenotype. In addition, in-vitro studies demonstrate that MSCs, driven by inflammatory signals, enhance phenotypic and functional changes on microglia/macrophages. In particular, MSCs, through the release of CX3CL1, may promote the switching of microglia from pro-inflammatory (type 1) to anti-inflammatory (type 2), and increase intracellular calcium concentrations and phagocytic activity [90]. Since SE leads to type 1 macrophages/microglia activation, MSCs may also contribute to reducing inflammation in this direction [82,91].

Neuroinflammation may induce different subtypes of reactive astrocytes, which exhibit a microglia-like phenotype, referred to as A1 (pro-inflammatory) and A2 (anti-inflammatory). The A1 astrocytes lack the main normal astrocyte function, being destructive to synapsis and neurotoxic to neurons [92].

Since astrocyte alterations are involved in the progression of epilepsy [93], protecting astrocyte from inflammatory stimuli could be a promising therapeutic strategy. In this regard, it has recently been demonstrated that MSC cell-derived exosomes could attenuate the A1 astrocytic activation in an animal model of TLE by regulating the Nrf2-NF-KB signaling pathway [94].

## 9. Perspectives

The simplicity in obtaining MSCs from both autogenic and allogenic sources, relative lack of serious adverse outcomes, and relatively non-invasive routes of administration make them attractive for preclinical and clinical applications. Indeed, the promising features of MSCs, in particular their immune-regulatory and anti-inflammatory properties, have raised strong interest among researchers in the field of epilepsy.

As has been demonstrated in various animal models of SE and chronic TLE, MSCs derived from multiple sources can reduce epileptogenesis and improve cognitive function, which raises the possibility of clinical applications, ideally including anti-epileptogenetic treatments in refractory *status epilepticus* and drug-resistant epilepsies. Therefore, the approach could overcome the shortcomings of currently available drug and surgical treatments.

Although preclinical studies have consistently shown the efficacy of MSCs in halting the progression of epilepsy, only two clinical trials have evaluated their effect in treating epilepsy in human patients. Both clinical trials are available on www.clinicaltrials.gov [95] but, as of this writing, final results have not been posted by either study. Standardization may represent a significant obstacle in obtaining consistent clinical trial results [96]. For example, cultured MSCs differ in behavior and marker expression, depending on species (mouse vs. human) and animal strains [97]. Given the importance of species differences for the significance of rodent-based studies as models of human disease, humanized MSCs have been created by incorporating the human IDO gene into MSCs derived from mice lacking the Nos2 (iNOS) gene (iNOS^−/−^). These humanized MSCs show immunomodulatory effects similar to those of normal human MSCs and thus represent a precious mouse model with which to mimic the human system [98]. Moreover, MSCs derived from different tissues have different growth abilities and phenotypic heterogeneity [99]. Finally, differences in funding and legislation might also be taken into account [100,101].

Therefore, to validate MSC-based therapy as a potential treatment for neurological disease, discussions on shared guidelines, cost coverage, and legislation support are required. Moreover, despite the quite extensive knowledge of in-vitro MSC functions, their in-vivo properties and potential clinical application have yet to be thoroughly investigated. To achieve this purpose, new specific markers allowing the in-vivo tracing of MSCs are required. Despite current limitations in clinical practice, in the last few years, several studies have provided new knowledge about the function of MSCs, especially within inflammation. It has become clear that the reparative ability of MSCs depends on the strength of the inflammation process. Therefore, MSCs may have different responses and growth-factor production to different levels of inflammation, cytokines, and immunosuppressants. Deepening our understanding of the plasticity of MSC-mediated immune regulation, together with the abovementioned adjustments, will help to improve the accuracy of MSC treatment, in order to achieve clinical relevance in the near future.

## 10. Conclusions

MSCs, as well as other types of stem cells, offer a potential tool for treating inflammatory diseases, including epilepsy. It seems that MSCs reduce epileptogenesis by means of trophic and anti-inflammatory factor release. Therefore, these secreted factors interact with cells of both immune and central nervous systems, contributing to restoring the neuronal homeostasis in affected areas. However, our knowledge is still inadequate for an immediate application of MSCs in clinical procedures for epilepsy’s treatment.

## Abbreviations

AMPA	α-Amino-3-hydroxy-5-methyl-4-isoxazolepropionic acid
BBB	Blood–Brain Barrier
BMMC	Bone Marrow Mononuclear Cell
DC	Dendritic Cell
GABA	γ-aminobutyric acid
GF	Growth Factor
ICAM-1	Intercellular Adhesion Molecule 1
Ig	Immunoglobulin
IL	Interleukin
IFN	Interferon
MSCs	Mesenchymal Stem Cells
SE	*Status Epilepticus*
TGF	Transforming Growth Factor
TLE	Temporal Lobe Epilepsy
Th	T helper
TNF	Tumor Necrosis Factor
VEGF	Vascular Endothelial Growth Factor
